# Innocuity of Milk from Tuberculous Cows

**Published:** 1912-10

**Authors:** 


INNOCUITY OF MILK FROM TUBERCULOUS COWS. Chausse, *Annales de l'Institut Pasteur,* July, 1911. The author considered 98% of bovine tuberculosis due to inhalation, except that in calves, subjected to risk from tuberculosis milk, 10% succumbed. He regards the practical danger of tuberculous cow's milk to human infants as slight.

